# Clinical Inertia in the Management of Type 2 Diabetes Mellitus: A Systematic Review

**DOI:** 10.3390/medicina59010182

**Published:** 2023-01-16

**Authors:** Turky H. Almigbal, Sarah A. Alzarah, Flwah A. Aljanoubi, Nouryah A. Alhafez, Munirah R. Aldawsari, Zahraa Y. Alghadeer, Abdullah A. Alrasheed

**Affiliations:** 1 Department of Family and Community Medicine, College of Medicine, King Saud University, Riyadh 12372, Saudi Arabia; 2 Department of Family Medicine, King Saud University Medical City, King Saud University, Riyadh 12372, Saudi Arabia; 3 Vision College of Medicine, Vision Colleges, Riyadh 13226, Saudi Arabia

**Keywords:** diabetes mellitus, type 2 diabetes, T2DM, clinical inertia, inertia

## Abstract

This review seeks to establish, through the recent available literature, the prevalence of therapeutic intensification delay and its sequences in poorly controlled Type 2 Diabetes Mellitus (T2DM) patients. The strategy identified studies exploring the clinical inertia and its associated factors in the treatment of patients with T2DM. A total of 25 studies meeting the pre-established quality criteria were included in this review. These studies were conducted between 2004 and 2021 and represented 575,067 patients diagnosed with T2DM. Trusted electronic bibliographic databases, including Medline, Embase, and the Cochrane Central Register of Controlled Trials, were used to collect studies by utilizing a comprehensive set of search terms to identify Medical Subject Headings (MeSH) terms. Most o the studies included in this review showed clinical inertia rates over 50% of T2DM patients. In the USA, clinical inertia ranged from 35.4% to 85.8%. In the UK, clinical inertia ranged from 22.1% to 69.1%. In Spain, clinical inertia ranged from 18.1% to 60%. In Canada, Brazil, and Thailand, clinical inertia was reported as 65.8%, 68%, and 68.4%, respectively. The highest clinical inertia was reported in the USA (85.8%). A significant number of patients with T2DM suffered from poor glycemic control for quite a long time before treatment intensification with oral antidiabetic drugs (OADs) or insulin. Barriers to treatment intensification exist at the provider, patient, and system levels. There are deficiencies pointed out by this review at specialized centers in terms of clinical inertia in the management of T2DM including in developed countries. This review shows that the earlier intensification in the T2DM treatment is appropriate to address issues around therapeutic inertia.

## 1. Introduction

Diabetes mellitus (DM) is a chronic global epidemic, a major health problem, and is one of the fastest growing global health emergencies of the 21st century. In 2021, 537 million people were estimated to have diabetes; this number is projected to reach 643 million by 2030 and 783 million by 2045. In addition, 541 million people were estimated to have impaired glucose tolerance (IGT) in 2021 [1]. Globally, 1.5 million deaths are directly related to diabetes each year [2], and in 2012, 3.7 million deaths were attributed to blood glucose levels directly or indirectly. Another 2.2 million deaths were due to increased risk of cardiovascular disease (CVD) and other diseases [3]. In this context, diabetes is the ninth leading cause of mortality worldwide [4]. Economically, it has been estimated that worldwide GDP losses will be USD 1.7 trillion due to diabetes from 2011 to 2030 [3].

Type 2 diabetes mellitus (T2DM) is the most common type of diabetes, accounting for over 90% of all diabetes cases [5]. The risk factors for T2DM include adiposity; genetic predisposition; unhealthy diet; low physical activity; smoking; certain biomarkers (e.g., raised alanine aminotransferase, C-reactive protein, and reduced vitamin D); and pre-existing medical conditions (e.g., hypertension, gestational diabetes, and preterm birth) [6]. The pathogenesis of T2DM involves the intricate interactions of genetic, metabolic, and environmental factors leading to beta-cell dysfunction, insulin resistance, and subsequent elevated blood glucose levels [7]. 

Glycemic control is critical in diabetic patients for T2DM management. If not strictly controlled, there can be serious diabetic complications, such as retinopathy (blindness), nephropathy (kidney failure), CVD, diabetic ketoacidosis (DKA), and a hyperglycemic hyperosmolar state (HHS) [8]. Clinical guidelines recommend the monitoring of HbA1c every three months with frequent therapy modification until the glycemic goal is achieved [5]. Despite the development of new medications over the past decades, a significant proportion of people with T2DM failed to achieve glycemic goal due to delayed stepping up in therapy by the healthcare providers [9]. In terms of optimal management of diabetes, clinical inertia is a remarkable barrier to therapeutic success, because it delays the intensification of treatment [10]. Therefore, clinical inertia may place a large proportion of patients at risk of experiencing suboptimal glycemic control for years before treatment is intensified. Therefore, this phenomenon is becoming highly recognized in T2DM management [11]. 

Clinical inertia is defined as “failure to initiate or intensify therapy according to the guidelines” [12]. In diabetes care, the difference between guidelines and clinical practice is defined as “clinical inertia” or “therapeutic inertia”. The concept represents the lack of appropriate modification or therapy ranging from lifestyle modification to the initiation of insulin therapy [10]. In about 30% of the individuals, IGT or impaired fasting glucose (IFG) progresses to diabetes. Thus, focusing on clinical inertia throughout the natural history of diabetes can help reduce this percentage by identifying any lack of intervention at multiple levels [10]. Recently, to avoid erroneous practice, a new definition of clinical inertia has been added to the literature. It defines clinical inertia as “the failure to start a therapy or its intensification/de-intensification when appropriate” [10]. 

Causes for therapeutic inertia are complex, multifactorial, and can be observed at three levels: the provider’s level, the patient’s level, and the system barriers level, which account for 50%, 30%, and 20% of therapeutic inertia causes, respectively [13]. At the provider’s level, lack of time, poor knowledge, varied or frequent guidelines, and fear of side effects such as hypoglycemia in diabetic patients are the major causes of therapeutic inertia. At the patient level, concerns over drug side effects, misunderstanding the different treatment regimens, comorbidities, terminal illnesses, trypanophobia, a limited doctor–patient relationship, low education level, and socioeconomic status are major barriers for therapeutic intensification [10]. At the system level, healthcare issues, high costs, poor communication and coordination between the departments, inadequate support technologies or insurance coverage, and regional differences of norms are some important causes of therapeutic inertia [13].

Studies show that inertia was remarkably higher in patients with mean glycated hemoglobin (HbA1c) close to the target (i.e., 7–8%) or those at the initial steps of diabetes management such as lifestyle modification and monotherapy [14]. A retrospective study conducted in Japan showed clinical inertia with real-world data. The study reported that approximately 50% of patients are above the HbA1c target levels for more than 6 months, regardless of how many oral antidiabetic drugs (OAD) they took [15]. In Thailand, 26.2% of patients with T2DM showed clinical inertia, with HbA1C levels ≥7.0% for periods ranging from 1.6 to 7 years, until intensification took place [16]. Early therapeutic intensification within three months showed a 1.36-fold higher likelihood of achieving the HbA1c goal when compared to late intensification within 10 to 15 months [17]. 

This review seeks to utilize the available literature, especially recently published studies, that cover the prevalence of clinical inertia and factors associated with it in poorly controlled T2DM patients. The literature is rich in studies that discuss the prevalence of therapeutic intensification delaying and its consequences on T2DM patients. The primary objective of our study was to identify studies exploring clinical inertia and its associated factors in the treatment of patients with T2DM.

## 2. Materials and Methods

**Figure 1:**
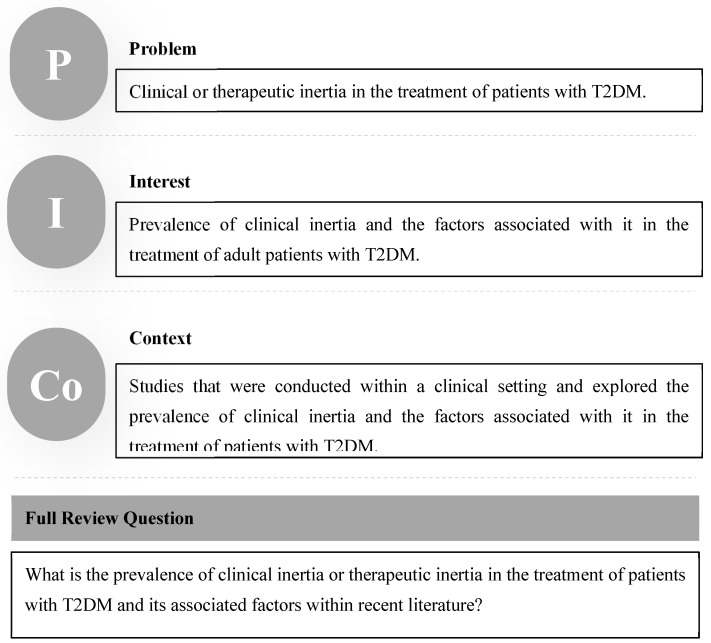
PICo elements (PICo framework).

This review used the PICo framework to define the study the review question. Each PICo framework element was defined (Figure 1). Following the PICo framework, the full review question was “What is the prevalence of clinical inertia or therapeutic inertia in the treatment of patients with T2DM and its associated factors within the recent literature?”

### 2.1. Study Type and Data Sources

This is a comprehensive systematic review conducted using trusted electronic bibliographic databases, including Medline, Embase, and the Cochrane Central Register of Controlled Trials. We collected studies that focused on any intervention addressing clinical inertia in the management of T2DM using a comprehensive set of search terms.

### 2.2. Search Strategy

A comprehensive set of search terms was developed using Medline to identify Medical Subject Headings (MeSH) terms related to our topic. We then identified text keywords based on our knowledge of the field (Table 1). The Medline search terms were modified for other electronic databases, including Embase and the Cochrane Central Register of Controlled Trials.

The search strategy identified both published and unpublished studies. A three-step search strategy was utilized in this review. An initial limited search of PubMed was performed, followed by an analysis of the text words contained in the title and abstract. The initial keywords used were T2DM OR T2DM management OR T2DM poor control or Type 2 DM clinical. The word “AND” was used to combine two concepts. A second search using all identified keywords and index terms was then performed across the newly added databases. Third, the reference list of all identified reports and articles was searched for additional studies.

### 2.3. Inclusion and Exclusion Criteria

The PICo framework led to the eligibility criteria (Table 2). This process included and defined both the inclusion and exclusion criteria.

The decision for the study inclusion in the review was based on matching the inclusion criteria (Table 3). Studies published in the mentioned databases from the date of their inception to the 31st of December 2021 were considered for inclusion in this review. The studies had to be published in English from any clinical setting that explored the prevalence of clinical inertia and the factors associated with it in the treatment of patients with T2DM. The inclusion criteria for participants were any patient ≥18 years, male or female, with T2DM. The studies that did not describe the factors associated with clinical inertia in the treatment of patients with T2DM, published in other than the English language, did not relate to the search question. Subjects aged <18 years were excluded from the review.

### 2.4. Data Extraction

**Figure 2:**
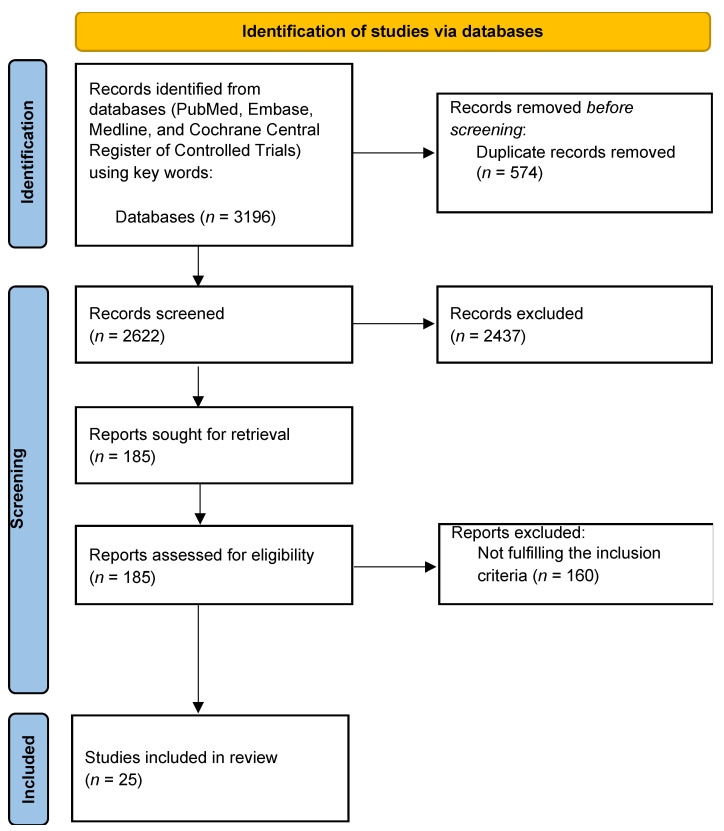
Flow chart of the search strategy using keywords (PRISMA diagram).

This review utilized the recommendations outlined in the Performed Reporting Items for Systemic review and Meta-Analyses (PRISMA) statement by following the PRISMA checklist and PRISMA flow diagram [18]. The title and abstract of searched-for studies were reviewed initially by two independent reviewers to ascertain that all included articles were in line with the inclusion criteria utilizing the PRISMA statement recommendations (Figure 2); these were then followed by a full-text review. All studies that explored clinical inertia in the treatment of patients with T2DM and described the associated factors were collected to extract data. The following information was gathered using a standardized form: authors, year of publication, location, study design, period, sample size, patient, physician, treatment intensification definition, glucose-lowering agents used before and after treatment intensification, and therapeutic inertia measures. HbA1c thresholds were used to identify patients who required treatment intensification.

### 2.5. Methodological Quality and Risk of Bias

The risk of bias in the studies was assessed by the Newcastle–Ottawa scale (NOS) [19], which consists of three domains: (1) selection, (2) comparability, and (3) assessment of the outcome(s). A score of 0–9 was allocated to each study. Studies with an NOS score ≥6 were considered high quality. If any disagreement arose between the independent reviewers regarding the search results and study inclusion, it was resolved by a third independent reviewer through discussion.

## 3. Results

### 3.1. Selection and Descriptive Assessment

The initial literature search identified 3196 studies, but this review concluded a final list of only 25 studies. The comprehensive databases (PubMed, Embase, Medline, and Cochrane Central Register of Controlled Trials) initial search identified 3196 studies, which contained 574 duplicate studies. Another 2437 studies had titles or abstracts only, which shortened the list to 185. Of these, 160 studies did not fulfill the defined inclusion criteria.

This review thus includes 25 studies (Table 4) published from 2004 to 2021. Only one study was published per year in 2019, 2013, 2012, 2010, 2005, and 2004. Two studies were published per year in 2021, 2020, 2015, and 2014. There were three per year in 2017 and 2016, with four in 2018.

The list thus contained different study designs. Fifteen studies used a retrospective design, four studies used a prospective design, five studies used a cross-sectional design, and one study used a qualitative study design.

The total number of T2DM patients covered by the list was 575,067 patients. Most patients were aged 60 years or above. Fourteen studies had more than 50% of male patients. Six studies were conducted in the United States of America (USA) and included 141,714 patients. Six were in the United Kingdom (UK) and included 214,516 patients. Spain and France each had two studies with 37,502 and 1971 patients, respectively. One study in the list was conducted internationally across six countries (including Brazil, India, Japan, Spain, the UK, and the USA) and had 652 patients. There was one study across both Germany and Australia with 4576 diabetic patients treated with dipeptidyl peptidase-4 inhibitors (DPP-4i) and/or sodium/glucose cotransporter 2 inhibitors (SGLT-2i). In Croatia (10,275 patients), Colombia (363 patients), Taiwan (168,876 patients), Thailand (98 patients), Brazil (323 patients), Malaysia (7646 patients), and Canada (379 patients), only one study was conducted.

The included studies interviewed a variety of healthcare practitioners, mainly family physicians. One of these studies interviewed 19 physicians and one nurse who had been involved in managing T2DM patients to identify and explore perceptions about clinical inertia from the perspective of the primary healthcare providers. Another study included 109 family physicians to determine clinical inertia existence in family medicine practice among patients with T2DM requiring insulin therapy.

### 3.2. Criteria for Clinical Inertia

The criteria for clinical inertia varied among the included studies, because it was studied in a variety of settings. The criteria for clinical inertia included poor glycemic control as indicated by mean HbA1c levels above the recommended target levels [20], failure to initiate or intensify treatment in a timely manner as indicated by a consultation in which treatment change based on HbA1c levels is indicated but does not occur [21], lack of individualization of treatment goals for some patients [22], failure to initiate or intensify treatment in accordance with evidence-based guidelines [25], and failure to initiate or intensify therapy when indicated [30].

### 3.3. Aspects of Clinical Inertia

The studies covered different aspects of clinical inertia. The aspects that were identified by this review included the prevalence of clinical inertia; the time to treatment intensification; the potential reasons for clinical inertia or treatment non-intensification among physicians and patients; the perceptions of primary care providers about clinical inertia; the patterns and predictors of treatment intensification; the effect of clinical inertia on glycemic control; and the factors associated with delayed treatment in patients with newly diagnosed T2DM, complications of T2DM, and comorbidities.

### 3.4. Clinical Inertia Prevalence

In most of the studies, the prevalence of clinical inertia was over 50% in patients suffering from T2DM. Although the prevalence was high in most of the included studies, it varied geographically depending on the country where the study was conducted. In the USA, clinical inertia ranged from 35.4% to 85.8% (average 60.6%). It was at a lower percentage in the UK, where clinical inertia ranged from 22.1% to 69.1% (average 45.6%). It was even lower in France and Spain, where clinical inertia ranged from 31.0% to 42.3% (average 36.6%) and 18.1% to 60.0% (average 39.0%), respectively. In countries including Canada, Brazil, and Thailand, clinical inertia was reported at prevalence percentages higher than 60.0% at 65.8%, 68%, and 68.4%, respectively. On the other hand, countries including Croatia, Colombia, Germany, Australia, Taiwan, and Malaysia reported clinical inertia at prevalence percentages lower than 60.0% at 55.7%, 56.9%, 55.6%, 38.3%, and 54.6%, respectively. The highest clinical inertia was reported in the USA (85.8%), and the lowest clinical inertia was reported in Spain (18.1%).

### 3.5. Clinical Inertia as Delayed Treatment Intensification

A significant number of patients with type 2 diabetes mellitus (T2DM) had poor glycemic control for extended periods of time before treatment intensification with oral antidiabetic drugs (OADs) or insulin was initiated. Khunti et al. [20] reported that the median time between initiation and intensification of treatment with additional OADs was more than seven years among patients on one, two, or three OADs. Lanzinger et al. [24] found that the average time in poor glycemic control was 12.6, 9.9, and 8.4 months for HbA1c levels above 7.0%, 7.5%, and 8.0%, respectively. Similarly, Romera et al. [25] reported a median time to first treatment intensification of 456 days or 15.2 months. Mata-Cases et al. [35] found a median time for treatment intensification of 17.1 months for HbA1c levels of 8.0–9.9% and 10.1 months for HbA1c levels above 10%.

### 3.6. Factors Associated with Clinical Inertia

Potential factors associated with the clinical inertia were explored within our list of studies. We sought to identify the reasons for clinical inertia or treatment non-intensification among physicians and patients. The identified factors included patient reluctance, a fear of side effects, a higher percentage of HbA1c, OAD started by diabetologist, patients using more than OADs, a longer duration of diabetes, an increased postprandial glycemia, total cholesterol, patient physical inactivity, communication gaps between the physicians and patients, older age, zero diabetes comorbidity severity index (DCSI), zero chronic illness with complexity (CIC) score, the use of calcium channel blockers (CCBs), a diagnosis of coronary artery disease (CAD), multimorbidity, and Black ethnicity. In contrast, a higher Charlson Comorbidity Index (CCI) score, Point-of-Service (POS) insurance, a high index HbA1c, a baseline endocrinologist visit, and the use of comedications were associated with a reduced likelihood of clinical inertia [34]. The review also came across two conflicting results, where Lang et al. reported that the patients with worse glycemic control and those whose therapy was initiated by a diabetologist experienced more clinical inertia [21], which contrasted with what Ziemer et al. reported, which was less clinical inertia at the diabetes clinic (35%) versus the medical clinic (67%) [43].

## 4. Discussion

This systematic review explored the prevalence of clinical inertia or therapeutic inertia and its associated factors in the treatment of patients with T2DM. This review also searched for potential reasons underlying a lack of intensifying treatments among physicians and patients along with exploring the perceptions of primary care providers about clinical inertia and predictors of treatment intensification outcome of clinical inertia on glycemic control and complications of T2DM.

Clinical inertia is detrimental to therapeutic success in the management of T2DM, and it would result in the development of complications [11]. Most of the included studies showed that considerable clinical inertia existed. Patients still showed suboptimal glycemic control despite the introduction of several glucose-lowering drugs in the management of T2DM.

There are multifactorial causes behind the clinical and therapeutic inertia, and this review has revealed several reasons for treatment non-intensification. Several factors can predict clinical inertia in the management of T2DM. Lin et al. [34] reported that old age, use of more than one OAD, use of CCBs, and above-target HbA1c predict a lower likelihood of intensification. However, they reported that a higher CCI score, POS insurance, high index HbA1c, baseline endocrinologist visit, and use of comedications predict the likelihood of treatment intensification or reduced clinical inertia. Mata-Cases et al. [35] reported that treatment intensification was associated with HbA1c of 8.0-9.9% or more than 10%, diabetes duration of ≥20 years, female gender, and presence of a comorbidity.

The values of HbA1c and duration of diabetes show conflicting results in different studies. For example, Khunti et al. [31] reported that a longer duration was associated with clinical inertia, while Mata-Cases et al. [35] reported that diabetes duration ≥ 20 years was associated with treatment intensification. Similarly, Lanzinger et al. [24] reported that a longer duration of diabetes was associated with treatment intensification. Similarly, the level of HbA1c role is also conflicting in different studies in terms of clinical inertia or treatment intensification. Clinical inertia results in poor glycemic control and speeds up the progression to complications such as diabetic retinopathy and reduced life expectancy [11,29].

Barriers to treatment intensification exist at the provider, patient, and system levels. To understand clinical inertia, barriers are needed at all the three levels. Zafar et al. [27] reported that most of the participants were unaware of the term “clinical inertia”. Patient education and exploring individual health beliefs may help in reducing clinical inertia. Clinical inertia results in poor glycemic control and speeds up the progression of diabetic retinopathy (shorter duration than that of non-inertia group).

Interestingly, at the provider level, some of the physicians admit their responsibility for clinical inertia because they think that health professional factors far outweigh the factors at the patients’ end. They also think that they should be a bit firmer to reach the target HbA1c [27]. Even some physicians defended their patients by referring to a lack of knowledge and taking the responsibility by not improving their patients’ awareness. This acknowledgement of responsibility should be regarded positively as a motivator for change. At the same time, some physicians directly blamed their patients for not complying with their instructions or not visiting their clinic on time [27]. Surprisingly, Zafar et al. [27] reported that most of the participants (physician or nurse) were unaware of the term “clinical inertia” or its meaning, but they could offer relevant explanations of the concept. This shows that the term clinical inertia or therapeutic inertia is rare, and it requires further work to put it side by side with clinical guidelines. In addition to the provider-to-patient relationship that was noted by this review, the provider-to-provider relationship has an important role to play in patient management as well. Interprofessional education (IPE) and interprofessional collaboration (IPC) have a positive impact on chronic disease management including T2DM, which results in quality-of-care improvement and an enhancement in the delivery of patient care [44].

At the patient level, barriers included non-compliance to prescribed medications, failure to attend appointments, conflicting priorities (such as perceptions, beliefs, or work commitments), lack of awareness about the fatality of T2DM, lack of symptoms, human error, comorbidities, polypharmacy, complex nature of the disease, deprivation, and communication barrier [27]. At the system level, barriers included time constraints, workload pressures, and lack of expertise. Surprisingly, in Croatia, Lang et al. [21] reported that patients whose therapy was initiated by a diabetologist experienced a worse glycemic control and higher clinical inertia than patients who were managed by other family physicians. This drawback at the level of diabetologists may be attributed to the problem in healthcare system based on poor monitoring and feedback on the specific outcome. This problem can be mitigated via tailored interventions such as educational courses, awareness sessions, peer influence, reminders, and even incentives. On the contrary, in the USA, Ziemer et al. [43] reported that patients who were treated at a diabetes clinic (or specialist clinic) experienced less clinical inertia than those who were managed at medical clinic. The difference in clinical inertia at the expert level between the two countries (Croatia and USA) may be attributed to the system integration levels where the US healthcare system is more integrated and well-coordinated.

Clinical inertia not only exists at the provider or patient level but also at the system level as well. Reasons for non-intensification include therapeutic failure, poor adherence to lifestyle modifications, poor compliance to medications, patient reluctance to intensify treatment, fear of side effects (such as hypoglycemia, gastrointestinal disturbance, oedema, or weight gain), poor self-monitoring, and cost [37]. Surprisingly the level of clinical inertia levels seen here were not related to health spending; for example, the highest clinical inertia was reported in a study conducted in the United States despite the high healthcare spending in USA [32]. A Spanish study showed the lowest clinical inertia [35]. 

This study shows that clinical inertia can have negative consequences for T2DM patients, which may contribute to poor prognosis. Low quality of care can further exacerbate these negative consequences. Low quality of care may result in visit-to-visit HbA1c variability among T2DM patients and relatively fewer outpatient visits. It may increase the risk of those patients receiving suboptimal preventive care for their diabetes [45,46]. A careful review of patients’ historical HbA1c measures and more high-quality preventive visits may help to identify patients at high risk and tailor their care accordingly.

Clinical inertia may seem to save money in the short term, but it can add costs in the long-term due to the negative consequences of uncontrolled disease. In Italy, the long-term projections of the Associazione Medici Diabetologi-annals initiative (a physician-led quality-of-care improvement program for T2DM patients) indicate that this initiative is a cost-saving method of improving clinical outcomes in T2DM patients because it improves HbA1c, blood pressure, lipid profiles, and BMI in enrolled patients compared to conventional management methods [47].

One of the strengths of this review is that it addresses the importance of clinical inertia in the management of T2DM. It also shows how clinical inertia can change across different health systems and countries.

As in most of the studies, this study does have some limitations. This review presents pooled study findings conducted across 14 different geographical areas and may be considered broadly representative of the T2DM clinical inertia. Limitations should thus be highlighted. This review lacks a quantitative approach. Future work with meta-analysis would contribute more to the understanding of clinical inertia among T2DM patients and would provide a deeper understanding on lessening the effect of clinical inertia on T2DM patient management.

Regarding COVID19 effects on patients, practitioners, and systems, this study could not address the effect of COVID 19 pandemic on T2DM clinical inertia. The studies used here were published between 2004 and 2021; only three studies were conducted in 2020 and 2021. This establishes a new opportunity to explore the clinical inertia more by conducting further studies to show, for example, the effect of the pandemic on this aspect of clinical practice.

## 5. Conclusions

Our review found that clinical inertia is a significant issue in the management of T2DM even at specialized centers. This problem remains despite the availability of newer antidiabetic drugs and the well-recognized negative consequences of poor glycemic control on diabetes complications. Potential strategies for addressing clinical inertia in the management of T2DM include patient education, exploring patients’ beliefs about treatment, improving healthcare providers’ expertise, and complying with evidence-based recommendations.

In conclusion, our review highlights clinical inertia deficiencies in the management of T2DM at specialized centers even in developed countries. The results emphasize the importance of the timely intensification of T2DM treatment to address the issue of therapeutic inertia and improve patient outcomes.

## Figures and Tables

**Table 1:** Medical Subject Headings (MeSH) terms.

No.	Term
1	exp Diabetes Mellitus, Type 2/
2	type 2 diabetes.mp.
3	(T2D ^1^ or T2DM ^2^ or NIDDM ^3^).tw
4	diabet * and ((non insulin * depend *) or (noninsulin * depend *) or (noninsulindepend *).tw
5	Insulin resistance/or (obes * adj2 diabet *).tw
6	1 or 2 or 3 or 4 or 5
7	insulin intensification.mp.
8	(insulin adj2 intensi *).tw.
9	(clinical adj2 inertia).tw.
10	(therapeutic adj2 inertia).tw
11	(treatment adj2 intensi *).tw.
12	((therapy or therapeutic) adj2 intensi *).tw.
13	7 or 8 or 9 or 10 or 11 or 12
14	6 and 13
15	limit 14 to humans

^1^ Type 2 Diabetes; ^2^ Type 2 Diabetes Mellitus; ^3^ Non-Insulin Dependent Diabetes Mellitus; * represents any group of characters, including no character.

**Table 2:** Studies’ eligibility criteria based on the PICo framework.

PICo	Inclusion	Exclusion
Problem	Studies address the clinical inertia in the treatment of patients with T2DM.	Studies that do not address the clinical inertia in treatment of T2DM patients.
Interest	Studies explored the prevalence of clinical inertia and the factors associated with it in the treatment of adult patients with T2DM and were published in English.	No clinical inertia prevalence or factors associated with it were addressed. Study’s population included patients younger than 18 years. Not published in English.
Context	Conducted recently, in any clinical setting, of any design, and explored the prevalence of clinical inertia and the factors associated with it in the treatment of T2DM patients.	Studies were not conducted in clinical settings.

**Table 3:** Studies’ inclusion criteria.

No.	Inclusion Criteria
1	The study should be published on 31 December 2021 or before to be included. Studies published after 31 December 2021 will not be considered for inclusion.
2	The language of the study must be English language; any study published in language other than the English language will not be considered for inclusion.
3	The study to be included should be conducted in a clinical setting.
4	The study aim should be the evaluation of clinical inertia prevalence, as well as the factors associated with it, in the treatment of T2DM patients.
5	The study participants must have a diagnosis of T2DM and be 18 years of age or older.

**Table 4:** List of studies included in this review.

Authors, Year, Country	Study Title	Study Design	Participants
Khunti et al. 2013 UK [20]	Clinical inertia in people with type 2 diabetes	Retrospective cohort study	81,573 patients with T2DM Male (45.6%) & Female (54.4%) Mean age: ±65 years
Lang et al. 2015 Croatia [21]	Family physician clinical inertia in glycemic control among patients with type 2 diabetes	Multicenter, observational, cross-sectional study	10,275 patients with T2DM Male (48.1%) & Female (51.9%) Mean age: ±65 years
Balkau et al. 2016 France [22]	Reasons for non-intensification of treatment in people with type 2 diabetes receiving oral monotherapy: Outcomes from the prospective DI Attitude	Prospective DI Attitude study	1212 patients with T2DM Male (58.5%) & Female (41.5%) Mean age: 68 years
Machado-Duque et al. 2017 Colombia [23]	Effectiveness and clinical inertia in patients with antidiabetic therapy	Cross-sectional study	363 patients with T2DM Male (46.5%) & Female (53.4%) Mean age: 62 years
Lanzinger et al. 2018 Germany, Australia [24]	Clinical inertia among patients with type 2 diabetes mellitus treated with DPP-4i and/or SGLT-2i	Multicenter, prospective study.	483,421 diabetic patients. Males (56.5%) & Females (43.5%) Age: ≥18
Romera et al. 2020 Spain [25]	Clinical Inertia in Poorly Controlled Type 2 Diabetes Mellitus Patients with Obesity: An Observational Retrospective Study	Observational, multicenter, retrospective study	13,824 diabetic patients Male (54.9%) & Female (45.1%) Mean age: 65.5 years
Strain et al. 2014 Six Countries [26]	Time to Do More: Addressing Clinical Inertia in the Management of Type 2 Diabetes Mellitus	Cross-sectional study	652 patients with T2DM Male (62%) & Female (38%) Mean age: 56 years
Zafar et al. 2015 UK [27]	Acknowledging and allocating responsibility for clinical inertia in the management of Type 2 diabetes in primary care: a qualitative study	Qualitative study	20 interviews (with 19 GPs and 1 nurse) managing T2DM patients
Huang et al. 2016 Taiwan [28]	Therapeutic inertia and intensified treatment in diabetes mellitus prescription patterns: A nationwide population-based study in Taiwan	Retrospective cohort study	168,876 patients with T2DM Male (45.84%) & Female (54.16%) Mean age: 60.5 ± 10.8 years
Osataphan et al. 2017 Thailand [29]	Clinical inertia causing new or progression of diabetic retinopathy in type 2 diabetes: A retrospective cohort study	Retrospective cohort study	98 patients with T2DM Male (40.8%) & Female (59.2%) Mean age: 62.3 ± 9.95 years
Alvarenga et al. 2018 Brazil [30]	Clinical inertia on insulin treatment intensification in type 2 diabetes mellitus patients of a tertiary public diabetes center with limited pharmacologic armamentarium from an upper-middle income country	Retrospective record-based study	323 patients with T2DM Male (39.3%) & Female (60.7%) Mean age: 65.8 ± 10 years
Wan et al. 2020 Malaysia [8]	Clinical inertia in type 2 diabetes management in a middle-income country: A retrospective cohort study	Retrospective cohort study	7646 patients with T2DM Male (39.5%) & Female (60.5%) Mean age: ±55 mean age
Khunti et al. 2016 UK [31]	Clinical inertia with regard to intensifying therapy in people with type 2 diabetes treated with basal insulin	Retrospective cohort study	11,696 patients with T2DM Male (55.7%) & Female (44.3%) Mean age: 65.5 ± 13.2 years
Seidu et al. 2017 UK [32]	Therapeutic inertia amongst general practitioners with interest in diabetes	Retrospective audit study	240 patients with T2DM Male (55.83%) & Female (44.17%) Mean age: 62.22 ± 12.10 years
Buysman et al. 2018 USA [33]	Glycaemic impact of treatment intensification in patients with type 2 diabetes uncontrolled with oral antidiabetes drugs or basal insulin	Retrospective cohort study	28,123 patients with T2DM Male (55.8%) & Female (54.2%) Mean age: 60.7 years
Lin et al. 2016 USA [34]	Does clinical inertia vary by personalized a1c goal? a study of predictors and prevalence of clinical inertia in U.S. managed care setting	Retrospective, observational study	79,805 patients with T2DM Male (58.3%) & Female (41.7%) Mean age: ±75 years
Mata-Cases et al. 2018 Spain [35]	Therapeutic Inertia in Patients Treated With Two or More Antidiabetics in Primary Care: Factors Predicting Intensification of Treatment	Retrospective study	23,678 patients with T2DM Male (53.5%) & Female (46.5%) Mean age: 66.7 ± 10.5 years
Harris et al. 2010 Canada [36]	Clinical inertia in patients with T2DM requiring insulin in family practice	Cross-sectional study	109 FPs (85% males) and their 379 patients with T2DM Mean age: 63.5 ± 12.8 years
Simon 2012 France [37]	Therapeutic inertia in type 2 diabetes: insights from the PANORAMA study in France	Observational, cross-sectional study	759 patients with T2DM Male (63%) & Female (37%) Mean age: 65.7 ± 11 years
Yam et al. 2013 USA [38]	Clinical inertia in type 2 diabetes: A retrospective analysis of pharmacist-managed diabetes care vs. usual medical care	Retrospective analysis study	113 patients with T2DM Males (35.2% PMCD & 62.% UMC) Females (64.8% PMDC & 37.3% UMC) Mean age: 50.1 ± 10.1 & 44.1 ± 9.4
Ruiz-Negron et al. 2019 USA [39]	Factors Associated with Diabetes-Related Clinical Inertia in a Managed Care Population and Its Effect on Hemoglobin A1c Goal Attainment: A Claims-Based Analysis	Claims-Based Retrospective Analysis study	3078 patients with T2DM Male (63.9%) & Females (36.1%) Mean age: 54.4 ± 10.6 years
Rattleman et al. 2021 USA [40]	A Retrospective Analysis of Therapeutic Inertia in Type 2 Diabetes Management Across a Diverse Population of Health Care Organizations in the USA	Retrospective analysis study	28,000 patients with T2DM Male (56.6%) & Female (43.4%) Mean age: 57.5 ± 10.1 years
Chudasama et al. 2021 UK [41]	Ethnic, social and multimorbidity disparities in therapeutic inertia: A UK primary care observational study in patients newly diagnosed with type 2 diabetes	Retrospective cohort study	120,409 patients with T2DM Male (54.2%) & Female (45.8%) Mean age: 63.5 ± 13.4 years
Grant et al. 2004 UK [42]	Clinical inertia in the management of type 2 diabetes metabolic risk factors	Prospective cohort study	598 patients with T2DM Male (49%) & Female (51%) Mean age: 67.5 ± 12 years
Ziemer et al. 2005 USA [43]	Clinical Inertia Contributes to Poor Diabetes Control in a Primary Care Setting	Prospective observational study	Patients with T2DM: 438 vs. 2157 Females: 76% vs. 68% Average age: 63 years vs. 59 years

## Data Availability

Not applicable.
